# Research progress on digestive disorders following traumatic brain injury

**DOI:** 10.3389/fimmu.2024.1524495

**Published:** 2024-12-20

**Authors:** Yongshuang Lin, Chengshan Hou, Cheng Wang, Rui Chen, Yunzhong Zhu, Qing Zhou, Binbin Shao, Yi Huang, Shun Li

**Affiliations:** ^1^ The First Affiliated Hospital, Guangxi University of Traditional Chinese Medicine, Nanning, China; ^2^ Trauma Center, The Second Affiliated Hospital of Nanjing Medical University, Nanjing, China; ^3^ Department of Neurosurgery, Jiangsu Integrated Traditional Chinese and Western Medicine Hospital, Nanjing, China; ^4^ Graduate school, Youjiang Medical College for Nationalities, Baise, China; ^5^ Department of Neurology, School of Medicine, University of Pittsburgh, Pittsburgh, PA, United States

**Keywords:** trauma, TBI, post-traumatic syndromes, gut-brain axis, digestive system disorders, neuroinflammatory, enteral nutrition

## Abstract

Traumatic brain injury (TBI) is a prevalent disease that poses a significant threat to global public health. Digestive dysfunction, as a common complication, is of particular importance to understand its pathogenesis, diagnostic criteria, and relevant treatment strategies. TBI can affect digestive function through inflammatory immune responses, the enteric nervous system, and hormonal levels. Furthermore, TBI can also impact neurologic recovery through bidirectional communication along the brain-gut axis. Therefore, this article aims to summarize the underlying mechanisms and further explore individualized feeding strategies, therapeutic approaches, long-term prognosis for TBI patients, as well as recent advancements in related technologies. Further understanding of the pathogenesis of digestive system dysfunction after TBI on the basis of the interaction of gut-brain axis is conducive to more future therapies to treat TBI and improve the long-term prognosis of patients through improving digestive function, and achieve good clinical efficacy.

## Introduction

Traumatic brain injury (TBI) refers to an acquired insult to the brain caused by an external mechanical force, which can lead to temporary or permanent impairment (1). In the United States, it is estimated that there are between 1.7 and 2.0 million cases of traumatic brain injury (TBI) occurring each year, resulting in approximately 5.3 million Americans currently experiencing long-term comorbidities linked to TBI (2). One comorbidity that is often overlooked following neurotrauma is dysregulation of the gastrointestinal (GI) organs associated with nutrient homeostasis (3). Patients suffering from gastrointestinal dysfunction frequently encounter alterations in the mucosal lining of their intestinal tract, an elevation in gut permeability, and irregularities in gut motility (4, 5). Following brain injury, intestinal epithelial cell dysfunction or apoptosismay occur due to ischemia and/or hypoxia, oxidative stress, and inflammatory reaction. These elements can ultimately result in inflammation, ulceration, and perforation. Intestinal epithelium curtains off the contents of the lumen, preventing the invasion of pathogenic antigens. Digestive dysfunction after TBI is also associated with higher mortality and frequent hospitalizations. With the increasing number of studies on the long-term prognosis of TBI patients in recent years, more studies have proved that digestive system disorders after TBI are related to cognitive function, depression, Parkinson’s disease and other diseases after injury. However, it is still a difficult problem to clarify the molecular mechanism of digestive system dysfunction after TBI, which has a crucial role in improving the long-term prognosis of TBI patients.

## Epidemiology and incidence of digestive disorders post-TBI

TBI is a chronic disease process characterized by persistent secondary injury processes which can be exacerbated by subsequent challenges (6). Digestive Disorders Post-TBI can also lead to related symptoms, such as gastrointestinal bleeding (7), gastroesophageal reflux (8), and decreased intestinal motility (9). These symptoms are mainly caused by damage to the mucosa of the digestive organs and changes in their movement patterns. One study showed that gastritis was found on esophagogastroduodenoscopy in 91% of patients (10). The gut microbiota, a vast collection of microorganisms within the human digestive tract numbering in the billions, performs a multitude of physiological roles. Disruption of the gut microbiota balance plays a pivotal role in the development of numerous localized and systemic disorders (11). Consequently, the permeability of the intestinal mucosa increases. TBI-induced intestinal permeability can cause the translocation of endotoxins and bacteria in the intestinal tract, further inducing or aggravating the systemic inflammatory response and resulting in multiple organ failure and death.

## Types and characteristics of digestive disorders

Recurrent injury to the gastroduodenal mucosa is frequently observed following a severe head trauma and manifests shortly after the incident. The major gastrointestinal changes observed after traumatic brain injury can be summarized in four aspects. Firstly, there is a reduction in blood supply to the gastrointestinal mucosa, leading to stress ulcers and bleeding in the digestive system (10). Secondly, there is a dysfunction in gut motility, resulting in symptoms such as abdominal bloating, diarrhea, delayed gastric emptying, and even intestinal paralysis (8, 12). Thirdly, there is disruption of the gut barrier function which allows bacteria and endotoxins to enter the bloodstream and contribute to systemic inflammatory response syndrome (SIRS) and sepsis. Lastly, there are alterations in nutrient absorption by the intestinal lining which can lead to malnutrition.

## Assessment and diagnosis of digestive disorders in TBI patients

At present, there is no unified standard for the diagnosis of digestive dysfunction after craniocerebral injury, which is mainly judged by history, clinical manifestations and related auxiliary examination. The diagnostic criteria for TBI are a clear history of trauma and confirmed as TBI by head CT or MRI. gastrointestinal injury (AGI) diagnosis criteria were evaluated according to the AGI grading system issued by the European Society of Critical Care Medicine in 2012 (13). In Zhang, D’s study, AGI score was applied to evaluate gastrointestinal functional impairment in critically ill patients (14). Although AGI score has shortcomings such as lack of objective indicators, it is still the most comprehensive method for evaluating gastrointestinal dysfunction in critically ill patients at this stage. Therefore, it can also be jointly diagnosed by laboratory and imaging examinations in clinical practice. Gastroparesis due to TBI or after nutritional support is diagnosed by bedside X-ray technique. Early identification and diagnosis of stress Cushing’s ulcer and clear source of gastrointestinal bleeding can be achieved through gastroscopy (15). Hsieh, JS ET al used gastroscopy to identify early ulcers and took endothelin-1 (ET-1), inducible nitric oxide synthase (iNOS) and macrophage inflammatory protein-1α (MIP-1α) from gastric mucosa to assess the degree of injury (16). Due to the increased intestinal permeability after TBI, harmful substances in the intestine may lead to further digestive dysfunction and even systemic inflammatory response. Hang, CH et al. Assessed intestinal barrier dysfunction by measuring the ratio of lactulose to mannitol (L/M) to determine serum endotoxin levels. Changes in fecal flora can also be used to indicate abnormal digestive system function. In the study of Nicholson, SE et al., the abundance of beneficial microbial phyla (e.g. Firmicteta) decreased when TBI patients were admitted, and opportunistic phyla (e.g. Proteobacteria) also decreased (*p* < 0.05) (17).

The routine use of dehydrating agents such as mannitol, anesthetic sedatives, and mild hypothermia in the treatment of the majority of TBI patients inevitably heightens the risk of developing digestive disorders (18). Moreover, studies substantiate that the prolonged application of mild hypothermia not only fails to mitigate the onset of secondary brain injuries following TBI but may also trigger gastrointestinal complications including dysfunction (19). Adike, A. et al. found in their research that gastrointestinal motility disturbances represent prevalent complications among patients in critical condition, significantly contributing to mortality rates. These complications frequently correlate with sepsis, mechanical ventilation, and the administration of vasopressors, opioids, or anticholinergic medications (20). In addition, treatment measures such as mechanical ventilation and the use of vasopressors also increase the risk of gastrointestinal bleeding and intestinal dysfunction (21).

## Mechanisms and pathophysiology of digestive dysfunction after TBI

Digestive disorders caused by TBI are mostly affected by the gut-brain Axis (GBA), a complex biphasic information exchange network between the Brain and the Gut (22).

**Figure 1:**
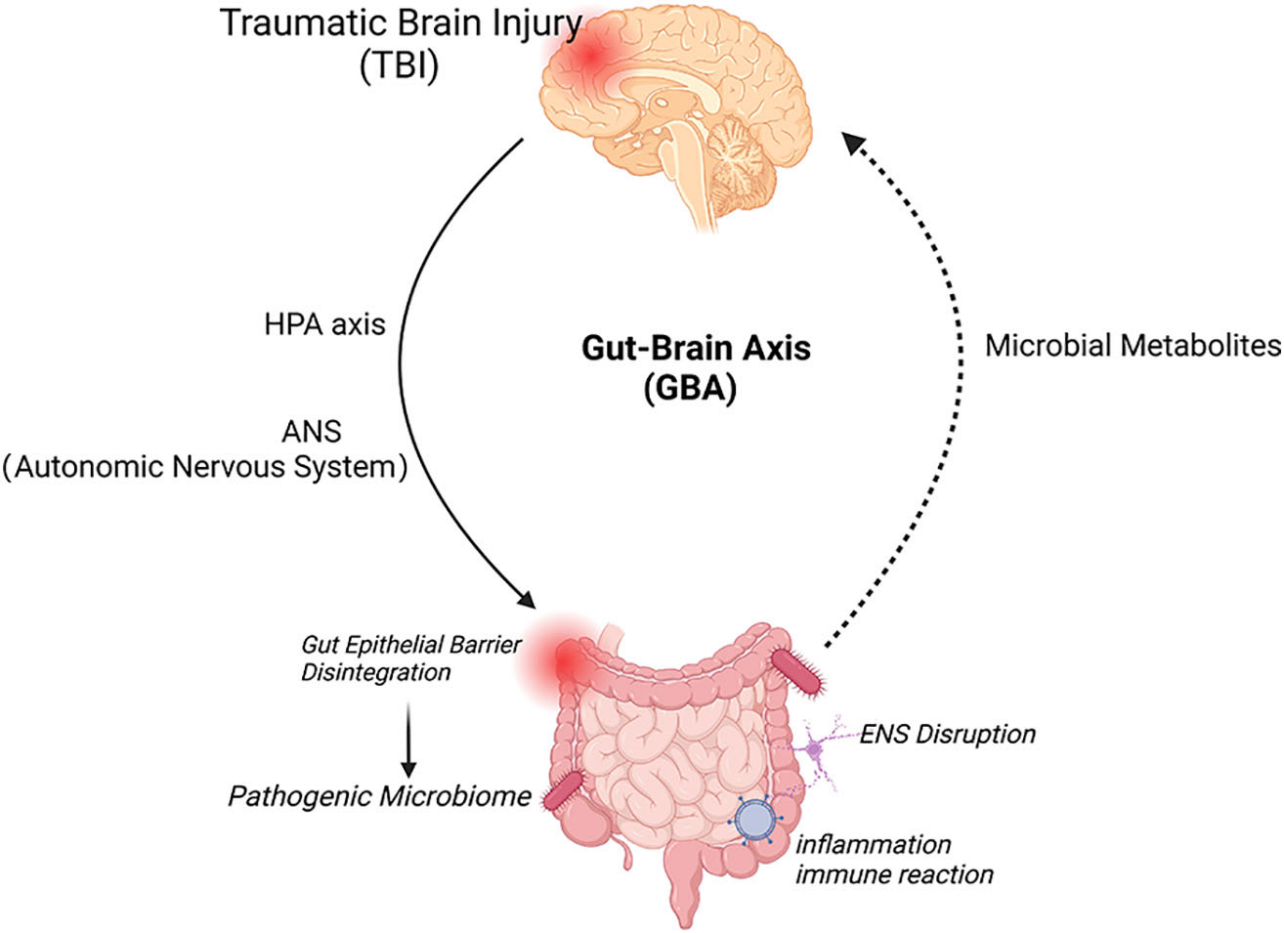
Bidirectional interactions of the brain-gut axis and therapeutic targets. The brain and gut communicate through direct (neurological) and indirect (systemic) two-way pathways. The brain, through the HPA axis and ANS (sympathetic and vagus efferent nerves), causes damage to the intestinal mucosa, including mechanical stimulation, bacterial displacement and other signals from the intestine, induces activation of local and systemic immune responses through ENS neurons, immune and endocrine mechanisms, increases the permeability of the blood-brain barrier and deteriorates the nervous system prognosis. Created with BioRender.com.

The gut-brain axis signaling pathway regulates digestive system function after TBI through the interaction of vagus nerve, intestinal microbiota, immune cell response, and neuroendocrine pathways (**Figure 1**).

(1) ENS(Enteric nervous system) can combine the unbalanced state of the sympathetic nerve and vagus nerve after TBI to further cause gastrointestinal damage (23). One of the main components of ENS ‘foreign nervous system is the vagus nerve,90% of which extends from the gut to the brain. There are a large number of dopamine neurons in the human digestive tract. The release of dopamine after TBI leads to sympathetic nerve excitation, and the inhibition of vagus nerve leads to digestive tract injury. Bansal, V et al. also confirmed that stimulation of the vagus nerve prevented TBI-induced increase in intestinal permeability in a mouse model of TBI by increasing the activity of glial cells (24).
(2) Changes in intestinal permeability and inflammatory response: TBI blood-brain barrier is destroyed, peripheral white blood cells are infiltrated (25), and immune responses are initiated in glial cells through cytokines, proteases and reactive oxygen species (26), leading to intestinal barrier disruption (4). Hang, CH et al. observed by histopathology and electron microscopy that the earliest intestinal mucosal damage occurred within 3 hours after TBI and lasted for 7 days (5). Results from Cannon AR et al. showed that, compared with healthy controls, TBI patients showed elevated levels of the systemic inflammatory cytokines IFN-γ and MCP-1, as well as elevated levels of IL-6 and IL-8 (*p* = 0.0551 and *p* = 0.0549) and the anti-inflammatory cytokine IL-4 Also decreased causing intestinal dysfunction resulting in increased tumor necrosis factor-α (TNF-α) in the gut and decreased bowel movement (27), This may be related to the fact that TNF-α not only induces damage to the close-knit structure of cells but also affects digestive system function by affecting the smooth muscle movement of the small intestine (28, 29). Research indicates that IL-13 is upregulated in human brain tissue and cerebrospinal fluid (CSF) within the first 24 hours following traumatic brain injury (TBI). A comparable increase in IL-13 and its receptor IL-13 Ra1 was observed in mouse models of TBI within 3 hours post-injury (30, 31).
(3) Changes of intestinal flora and hormone levels: With the increase of intestinal permeability and the damage of intestinal mucosa, intestinal flora also began to change, and the changes, translocations and diversity disorders of intestinal flora (reducing symbiotic bacteria and increasing the presence of pathogenic bacteria) induced a series of inflammation and immune responses by disrupting the bidirectional brain-gut axis balance, thus further aggravating gastrointestinal injury (32). It can even cause systemic inflammatory response syndrome (SIRS) (33). Cannon AR’s study also found that TBI significantly increased the number of copies of the patient’s potential pathogen Bilophila wadsworthia, thereby affecting the expression of inflammatory factors affecting the patient’s digestive function. At the same time, intestinal flora and many of its metabolites can lead to rapid and long-term inflammatory response through the secretion of pro-inflammatory lipopolysaccharides, further aggravating digestive disorders and poor prognosis (34, 35). TBI causes elevated hormone levels and can lead to long-term endocrine dysfunction. A multicenter prospective study by Chen, X et al. noted differences in the incidence of gastrointestinal bleeding and 28-day mortality associated with corticosteroids during the subacute phase of TBI (36). The HPA axis and GBA axis can also affect the secretion of gastrointestinal hormones such as somatostatin, further leading to digestive system dysfunction. Future studies are needed to further explore these mechanisms, especially the interactions at the molecular level, to provide new targets for the treatment of TBI-related digestive disorders.

## Management and treatment approaches for TBI-related digestive disorders

After TBI, due to various complex pathophysiological mechanisms, gastrointestinal motor dysfunction will lead to gastrointestinal symptoms such as vomiting, abdominal distension, diarrhea, gastric retention, and even toxic intestinal paralysis. Improving gastrointestinal motivity is the main direction of drug therapy. At present, the drugs used to improve gastrointestinal motivity mainly include metoclopramide, domperidone and Cisapride (37). Both metoclopramide and domperidone are dopamine receptor blockers, which can affect the central neuroendocrine function at the same time, but cisapride has no effect on neuroendocrine. However, with the extensive use of cisapride, problems such as prolonged Q-T interval and torsion-tip ventricular tachycardia can occur, and it is now replaced by the safer fourth-generation gastroenterokinetic drug moxapride. Komura, M., showed that mosapride reduced the incidence of gastroesophageal reflux in patients with neurologic impairment (38). In addition, there are a number of newly developed gastrointestinal motility drugs on the rise,

In recent years, Elmokadem, EM et al. proposed that the ratio of itopride to metoclopramide group could significantly increase the proportion of enteral nutritional feed, calories and protein in patients after one week (*p* = 0.001), (*p* = 0.002), (*p* = 0.01) (39). Whether erythromycin derivative therapy enhances gastrointestinal motility remains controversial (40, 41). In addition to gastrointestinal motivity drugs, it is also important to protect gastric mucosa, representative drugs are mainly omeprazole (OM), OM can effectively reduce the severity of stress ulcer in TBI patients by reducing the expression rate of ET-1 in tissues, iNOS and MIP-1alpha activity (16). Zhao, ZL study showed that TBI patients taking short-term high doses of glucocorticoids had an increased risk of death and a clear trend of clinical improvement compared with patients taking long-term low doses of glucocorticoids (42, 43). Ozay, R’s study also confirmed that omeprazole and methylprednisolone were equally effective in protecting the brain from oxidative stress and early apoptosis of TBI cells (44). The study by Palm, Nicole M, et al. suggests that trauma patients at risk of stress gastropathy can discontinue preventive medications once they tolerate enteral nutrition (45), so early enteral nutrition is a top priority for TBI patients.

TBI patients are generally at risk of poor eating or disorder, high energy consumption, digestive system dysfunction, high aspiration risk, abnormal glucose and lipid metabolism, intestinal flora disorders, and poor gastrointestinal tolerance, etc. Poor nutrition management will increase the risk of poor prognosis (46). Yang, L’s study pointed out that nutritional support for TBI patients can effectively shorten the length of hospital stay, reduce the infection rate, and play a positive role in promoting the rehabilitation of TBI patients (47). Parenteral nutrition (PN) and enteral nutrition (EN) are two methods mainly used for nutritional support. Qin, Y et al. found that the use of PN was more likely to induce peptic ulcer by comparing EN and PN. Chiang, YH pointed out that compared with EN patients, the risk ratio of non-EN TBI patients was 14.63 (95% CI 8.58-24.91), which may be similar to that of EN patients (48). This may be related to the fact that EN can increase gastrointestinal blood flow, restore nitrogen balance in the body, repair intestinal mucosal barrier structure, improve gastrointestinal motivity, reduce intestinal flora and endotoxin translocation, and improve immune function (49). Therefore, in recent years, EN, especially early EN, has attracted more and more attention due to its advantages of simplicity, convenience, low economic cost, and good effect and benefit, and has become the first choice for nutritional support for TBI patients (50, 51). Early EN refers to enteral nutrition treatment carried out within 24-48h of admission, and EEN is also recommended in ESICM Clinical Practice Guidelines published in 2017 (52). The study of Chourdakis, M showed that compared with delayed enteral nutrition, early EN can have a beneficial effect on hormone levels in patients with TBI, thereby improving prognosis (53).

Interdisciplinary collaboration has been shown to have a positive impact on the recovery of gastrointestinal function in patients with TBI. According to a number of studies, combined intervention using multidisciplinary teams (including neurology, rehabilitation, and nutrition) can significantly improve gastrointestinal dysfunction in patients with TBI, reduce the incidence of gastrointestinal complications, and improve overall survival. The Department of Nutrition can maximize the recovery of intestinal barrier function, optimize immune function, reduce inflammatory response, and improve overall prognosis by adopting a personalized feeding regimen in enteral nutrition. Studies have shown that personalized enteral nutrition programs reduce the incidence of gastrointestinal complications and shorten the length of hospital stay in patients with TBI (54).

The addition of various nutrients to enteral nutrition is also particularly important in improving digestive function in TBI patients (55, 56). In the study of Zhang, X, adding probiotics to enteral nutrition to treat TBI rats found that it could reduce the expression of dopamine receptor (DRs) in intestinal mucosa and prevent damage to intestinal mucosal barrier (IMB) (57). Sun, B et al. used Lactobacillus acidophilus to restore damaged Cajal interstitial cells (ICC) and damaged ICC network in TBI mice to prevent digestive disorders such as TBI-mediated inhibition of intestinal smooth muscle contractions (58). The Yi LJ study showed that supplementing EN with probiotics in early enteral nutrition effectively reduced the risk of infection, mortality, and gastrointestinal complications (59). Karakayal, EM et al. significantly improved outcomes in rats by reducing oxidative stress, apoptosis and gliosis and increasing vascular distribution through probiotic treatment (60). Cui yang et al. can induce immune tolerance by increasing Treg differentiation through oral brain protein combined with probiotics, thereby reducing secondary inflammatory injury after craniocerebral injury (61). Studies have shown that adding omega-3 fatty acids, curcumin, resveratrol, apigenin, vitamins and minerals to enteral nutrition can repair intestinal function, and ketogenic diet can improve the prognosis of patients with TBI (62). The addition of dietary fiber (DF) in enteral nutrition can produce short chainratty acids (SCFAs) under the action of human intestinal flora, thus improving digestive tract symptoms such as constipation and diarrhea in patients (63). The study of Yagmurdur, H also supports that dietary fiber should be increased while EN. Kurtz, P showed that multimodal monitoring of nutrition therapy, blood glucose control and brain microdialysis (CMD) could be used as an integrated approach to better manage TBI patients during enteral nutrition and diet adjustment.

After TBI, the brain stem, cerebellum and thalamus are damaged or intracranial high pressure leads to the inability to complete swallowing normally, resulting in swallowing disorder. In addition, dysconsciousness and cognitive decline in TBI patients may also affect swallowing function. The characteristics of traditional food do not fully meet the requirements of swallowing disorder patients, cannot cooperate with swallowing related rehabilitation training, daily meals are easy to cause coughing and aspiration. Therefore, relevant studies suggest that food traits can be changed to establish a safe and scientific diet training suitable for patients with dysphagia (64). At present, the main mainstream dysphagia training program is the International Dysphagia Diet Standardization Initiative (IDDSI), which was formulated in 2015 for thickening liquid food and daily rehabilitation training for patients with dysphagia disorders (65). Su, M et al. confirmed the feasibility of IDDSI framework in clinical and bedside applications by conducting a trial on 26 patients with dysphagia (66). An, S et al. further elaborated that the type of thickening thing seems to have more influence on the recovery of swallowing function (67). In addition to thickening liquid, some studies have pointed out that the recovery of swallowing function is also affected by the taste of sour, sweet, bitter and hot (68).

The joint rehabilitation department can improve the prognosis of patients through rehabilitation. Xing, X et al. used electroacupuncture to treat TBI patients and found that GIF, D-lactic acid (D-lac), diamine oxidase (DAO), lipolysaccharide (LPS), IAP and abdominal circumference were all lower in the acupuncture group at day 7, and compared with day 1 and day 3, The changes of the above indexes were similar (all *p* < 0.05). Therefore, early electroacupuncture treatment can improve digestive disorders in TBI patients. Therefore, patients with TBI need to work together with neurologists, gastroenterologists, rehabilitation doctors, dietitians, psychiatrists, etc. It is also very important for the neurosurgery department to evaluate whether early enteral nutrition can be implemented, the nutrition department to provide personalized nutritional meals, and the rehabilitation department to cooperate with the swallowing function and other rehabilitation exercise psychiatrists to pay attention to the psychological state of patients and provide necessary psychological support, so as to jointly achieve the goal of helping patients to obtain the best treatment effect and quality of life (48, 69).Therefore, promoting collaboration among interdisciplinary teams will be an integral part of TBI therapy in the future.

## Prognosis and long-term outcomes of digestive disorders post-TBI

The recovery of digestive system after TBI is affected by various factors, the first of which is individual differences. Weijun Fu’s study showed that female, severe GCS score, frontal injury, abnormal blood sodium, pulmonary infection, and intracranial infection are risk factors for recovery of digestive system disorders. Digestive dysfunction in people with TBI is not a short-term consequence, but rather a long-term one, and increases in incidence and severity over time (70). The majority of patients admitted to TBI for one year of rehabilitation services detected H. pylori infection. A 1-year follow-up of TBI with digestive disease found that the mortality rate was about 3 times higher than that of normal patients (71). Lower digestive tract dysfunction is a long-term and serious complication after TBI. The strong and rapid inflammatory response after TBI is not limited to the brain. With the increase of intestinal permeability, harmful substances such as intestinal bacteria enter the blood and lymphatic circulation, which can induce systemic inflammatory response and even threaten life. Mercado, NM et al., through a mouse model, found that TBI can increase systemic inflammatory response through peripheral blood albumin levels (72). Faries, PL et al. found that changes in intestinal permeability after severe trauma can further affect systemic inflammatory response syndrome (SIRS) and multiple organ failure (MOF) in patients (73). Finally, it is very likely that TBI is not a threat to patients’ lives, but the systemic inflammatory response and MODS induced by digestive disorders (74, 75).

On the other hand, digestive disorders can aggravate the original TBI brain injury and even cause more serious chronic neurological damage (76). Therefore, with the continuous improvement of people’s requirements for quality of life, most studies not only focus on early gastrointestinal and digestive disorders after DTI, but also focus on long-term longitudinal studies (77, 78). Celorrio, M., demonstrated in the study that antibiotic exposure 1 week after TBI reduced cortical infiltration of Ly6Chigh monocytes, increased microglial pro-inflammatory markers, and decreased T-lymphocyte infiltration, and that this effect could persist 1 month after injury. At the same time, the dysfunction of gut microbes can seriously aggravate the loss of neurons in the injured hippocampus and reduce the activation of microglia, which is accompanied by changes in the fear memory response (79), which may be related to the occurrence of mental diseases such as depression and cognitive disorders. li beixu et al. retrospectively analyzed 1027 cases of craniocerebral trauma caused by traffic accidents. The severity and area of craniocerebral trauma were closely related to the incidence of organic personality disorders (80). Studies by Chiu, LS et al. indicate that the incidence and severity of TBI is associated with an increased susceptibility to developing neurodegenerative diseases such as Parkinson’s or Alzheimer’s disease (81). So treatment requires long-term care and rehabilitation for the cognitive impairment and other clinical symptoms that people with TBI may experience. **Table 1** summarizes animal experiments, clinical studies, and cohort studies on the effects of TBI on digestive system dysfunction.

**Table 1:** Influence of traumatic brain injury on digestive dysfunction.

References	Study	Method	Results	Conclusion
Chen, X (36)	Experimental	A multicenter, prospective cohort study was conducted to evaluate the function of the hypothalamic-pituitary-adrenal axis in the subacute traumatic brain injury stage and the incidence of disease-associated corticosteroid hormone deficiency.	In the subacute stage of craniocerebral injury, disease-related corticosteroid hormone deficiency is common and strongly associated with poor prognosis.	Subacute infection of acute traumatic brain injury may be an important factor in the occurrence and development of corticosteroid hormone deficiency associated with severe disease.
Chiang, YH (48)	Experimental	Between 2002-2010, data were collected on 145 EN patients who received appropriate calories and nutrients within 48 hours after trauma and were compared with 152 non-EN controls matched with sex, age, weight, initial GCS score, and surgical status.	After adjusting for age, sex, initial GCS score, and recruitment period, the hazard ratio for non-EN patients compared to EN patients was 14.63 (95% CI 8.58-24.91). Among EN patients with a GCS score of 6-8, GCS scores improved significantly over the first seven ICU days compared to EN patients with a GCS score of 4-5 and non-EN patients with a GCS score of 6-8.	EN within 48 hours after injury was associated with survival, GCS recovery, and outcomes in patients with sTBI, especially those with a GCS score of 6-8.
Harrison-Felix, C (71)	Experimental	Retrospective cohort study.SETTING: Utilized data from the TBI Model Systems National Database, the Social Security Death Index, death certificates, and the US population age-race-gender-cause-specific mortality rates for 1994.	digestive conditions, and all external causes of injury/poisoning than were individuals in the general population of similar age, gender, and race.	Long-term follow-up of individuals with TBI should increase vigilance for, and prevention of, diagnoses frequently causing death (circulatory disorders) and diagnoses with a high relative risk of causing death in this population (seizures, septicemia, respiratory and digestive conditions, and external causes of injury).
Faries, PL (73)	Clinical trial	The IP of 29 patients with consecutive injuries requiring admission to a surgical intensive care unit and 10 healthy controls were measured using metabolically inactive markers ltose (L) and mannitol (M). Measurements were taken within 24 hours of admission and on the fourth day of hospitalization.	Patients with a significant increase in IP had a significantly increased chance of developing SIRS and subsequent infectious complications, and were strongly associated with multiple organ dysfunction scores.	Large increases in intestinal permeability are associated with the development of SIRS, multiple organ dysfunction, and an increased incidence of infectious complications.
Li, B (80)	Clinical trial	According to the International Classification of Diseases (ICD-10), 1 027 patients with craniocerebral injury caused by traffic accidents were retrospectively analyzed, and the degree of craniocerebral injury was classified and diagnosed as personality disorder after craniocerebral injury. The personality characteristics of all patients were evaluated using the simplified neuroticism, extraversion and openness five-factor scale (NEO-FFI).	The incidence of organic personality disorder after all kinds of traumatic brain injury was 33.1%, the incidence of organic personality disorder in moderate and severe traumatic brain injury patients was 38.7% and 44.2%, respectively, which was significantly higher than that in mild traumatic brain injury patients (18.0%) (P < 0.05).	The severity and size of craniocerebral trauma are closely related to the incidence of organic personality disorder, and also affect the clinical manifestations of the latter, which provides certain significance and help for forensic psychiatric identification.
Katzenberger (74),	Cohort	Levels of TNF-α and IL-6 in the brain were measured at 4 hours after TBI/UH, as well as levels of PARP-1 in the cortex that were cleaved.	ghrelin reduced brain levels of TNF-α and IL-6, reduced levels of the cleaved PARP-1 cortex, improved sensorimotor and reflex function, and reduced mortality after TBI/UH.	ghrelin has a great potential to be further developed as an effective resuscitation approach for the trauma victims with brain injury and severe blood loss.
Sun, Bo (58)	Cohort	To explore the molecular mechanism of probiotic Lactobacillus regulating intestinal motility in a mouse model of traumatic brain injury (TBI)	Treatment of TBI mice with Lactobacillus acidophilus significantly improved end-ileum villi morphology, restored damaged Cajal interstitial cells (ICC) and disrupted ICC networks after TBI, and prevented TBI-mediated inhibition of contractile activity of intestinal smooth muscle.	It provides a new mechanism basis for the application of Lactobacillus acidophilus in TBI therapy.
Wei, C (89)	Cohort	We evaluated the protective effect and possible mechanism of Tongqiao Huoxuestang in the treatment of gastrointestinal dysfunction induced by TBI by genetic engineering, histological staining, immunofluorescence (IF), 16S ribosomal ribonucleic acid (rRNA) sequencing, real-time polymerase chain reaction (RT-PCR) and enzyme-linked immunosorbent assay (ELISA), Western blot (WB) and flow cytometry (FCM).	TQHXD administration improved gastrointestinal dysfunction caused by TBI by regulating the abundance and structure of bacteria. Reconstruction of damaged IMB epithelium and chemical barrier; The ratio of M1/M2 macrophages, T regulatory cells (Treg)/T helper 1 cells (Th1) and Th17/Treg was improved to maintain the homeostasis of the intestinal immune barrier.	By regulating the intestinal biological, chemical, epithelial and immune barriers of IMB, the decoction plays a therapeutic role in the treatment of gastrointestinal dysfunction caused by TBI, which is generated by stimulating CD36/NR4A1/15-LO signaling pathway. However, it cannot do so when CX3CR1 and CD36 are defective. Therefore, TQHXD may be a potential candidate to treat gastrointestinal dysfunction caused by TBI.
Bansal, V (24)	Cohort	Balb/c mice receive weight loss TBI. Selected mice received electrical stimulation of the vagus nerve in the neck before TBI. Intestinal permeability to 4.4 kDa FITc-glucan was measured 6 hours after injury. The ileum was collected and intestinal tumor necrosis fact-α and glial fibrillary acidic protein (GFAP), a marker of glial activity, were measured.	Compared with vagus nerve stimulation + TBI, intestinal tumor necrosis factor-α increased 6 hours after injury in TBI animals (45.6 +/-8.6 pg/ml and 24.1 +/-1.4 pg/ml; p < 0.001).	In a mouse model of TBI, vagus nerve stimulation prevents TBI-induced intestinal permeability. In addition, vagus nerve stimulation can increase intestinal glial cell activity, which may represent a pathway for the central nervous system to regulate intestinal permeability.
You, W (32)	Cohort	We established TBI in mice using a lateral fluid strike damage model. Intestinal flora was examined by 16S rRNA sequencing, and bile acids were analyzed by ultra-high performance liquid chromatography with tandem mass spectrometry.	TBI causes intestinal inflammation and damage to the intestinal barrier. The changes of intestinal flora and bile acid spectrum were observed. From 1 hour to 7 days after injury, the diversity of intestinal flora underwent time-dependent changes. Bile acid levels in stool and plasma are reduced after TBI, and the decline in secondary bile acids is even more significant, which can lead to intestinal inflammation. Specific groups of bacteria that may cause changes in bile acid metabolism, such as Staphylococcus and Lactobacillaceae, were identified.	Tbi-induced intestinal flora disturbance may lead to gastrointestinal dysfunction by altering the bile acid profile. Intestinal flora may be a potential therapeutic target for TBI-induced gastrointestinal dysfunction.
Feighery, L (4)	Cohort	After craniotomy over the left medial prefrontal cortex on anesthetized rats, neurotrauma was produced using a pneumatically driven impactor on the exposed brain. Control rats were subjected to identical procedures but did not receive an impact. Muscle-stripped rat intestinal ileal and colonic segments were mounted in Ussing chambers within 30 minutes of death. Transepithelial electrical resistance (TEER) and the apparent permeability coefficient (Papp) of [C]-mannitol were recorded from intestinal tissue for 120 minutes. Histopathologic analysis was also performed to determine any gross morphologic changes in the intestine.	Ileal and colonic mucosae showed no differences in TEER in ileum or colon of TBI rats compared with controls. The Papp of mannitol was significantly increased in ilea from rats previously exposed to TBI compared with controls. Histologic analysis showed gross changes to 50% of the ileal but not the colonic sections from TBI rats.	TBI results in significantly reduced ileal barrier function, most likely mediated by open tight junctions. For patients with acute head injury, this may have implications for subsequent oral absorption of nutrients. Systemic delivery of luminal endotoxins may contribute to multiple organ failure.

## Current challenges and future directions in TBI-related digestive research

At present, the molecular mechanism of digestive system dysfunction induced by TBI is still not fully understood. In particular, the gut-brain axis (GBA), intestinal flora and systemic inflammation have not been fully explored. In the future, by utilizing high-throughput sequencing, gene-editing techniques, immune response and metabolic pathways, as well as animal models and clinical validation, more can be revealed about the molecular mechanisms by which TBI affects digestive function.

The diagnostic criteria for TBI-related digestive dysfunction are still inconsistent, which brings certain difficulties to clinical diagnosis and treatment. Most of the existing studies on TBI-related digestive dysfunction focus on the early stage after craniocerebral injury. There are still significant challenges in areas such as precision treatment strategies for TBI patients and the collection of long-term clinical data, and by filling these research gaps, we can provide more effective treatment options for TBI patients and mitigate the long-term impact of digestive complications on patient health.

In recent years, more and more studies have begun to focus on the neurological degeneration and psychiatric problems caused by long-term digestive disorders. Long-term follow-up data will help to better understand the natural history and prognosis of this complication. However, in Fang, Y’s study pointed out that the effective new techniques and methods required by IDDSI were not applied in time to patients with feeding difficulties after TBI (82). Therefore, cooperation is still needed in drug therapy, rehabilitation training, nutritional support and other aspects, and clinical application is still facing great challenges. Since the occurrence and presentation of digestive disorders after TBI vary from individual to individual, it is necessary to develop an individualized treatment plan for each patient’s specific situation. First, the individualized nutritional formula has more significant advantages than the conventional nutritional formula for TBI patients, which can reduce inflammation, improve the immune level of patients, improve intestinal mucosal barrier function, and have good gastrointestinal tolerance and low incidence of adverse reactions (83). Secondly, while improving the efficacy of treatment and the satisfaction of patients, individualized treatment helps to improve the self-management ability of patients, so as to participate more actively in the treatment process and better improve the prognosis (84).

Related studies have shown that fecal microbiota transplant-mediated Ghrelin recovery improves neural function after TBI by zhang yamei et al. (85). Berry, JAD et al. improved TBI patients’ ability to recover normal intestinal function and defecation through mesenteric elevation. Young, PJ, et al., reduced in-hospital mortality in TBI patients with selective digestive tract purification (SDD) (86). Traditional Chinese medicine treatment can also be used to effectively improve digestive disorders in TBI patients (87, 88) Wei, C The use of ventilation Huoxuet Decoction (TQHXD) in the treatment of TBI mice showed that TQHXD could significantly improve the differentiation of cluster 36 (CD36)/15-lipoxygenase (15-LO)/nuclear receptor subfamily of 4 group A members 1 (NR4A1) signaling is expressed in mouse colon tissue to improve digestive dysfunction in TBI mice (89). Baudo, G developed in mice a Lipo-Dex (liposome nanocoliths coated with dexamethasone) that selectively targets the injured brain, thereby reducing lesion volume, cell death, astrogliosis, release of pro-inflammatory cytokines, and activation of microglia, thereby improving prognosis (90).

## Conclusion

This paper discusses the relevant pathophysiology, diagnostic methods, and management strategies for digestive disorders following TBI. Future research is expected to further clarify diagnostic criteria and pathogenesis, particularly within the context of the GBA. This may offer new insights for clinical treatment of these disorders (91, 92). Effective treatment requires a multidisciplinary approach to develop more personalized plans (35), with clinicians making precise diagnoses based on specific patient symptoms and signs, supplemented by laboratory tests and imaging studies. An individualized treatment plan that includes medication, nutritional support, and rehabilitation training should be developed. Early and accurate assessment of TBI patients is crucial to ensure optimal recovery and minimize complications such as digestive disorders (93). Additionally, ongoing care measures can further support the rehabilitation of TBI patients post-discharge, helping to monitor long-term gastrointestinal dysfunction and other related issues. This is vital for the comprehensive study of TBI patients (94). In summary, future collaborative, multidisciplinary research aimed at addressing this complication could reduce mortality rates in TBI patients and enhance their quality of life.
